# Transitions in mental health and addiction care for youth and their families: a scoping review of needs, barriers, and facilitators

**DOI:** 10.1186/s12913-023-09430-7

**Published:** 2023-05-10

**Authors:** Roula Markoulakis, Hinaya Cader, Samantha Chan, Sugy Kodeeswaran, Tracey Addison, Cathy Walsh, Amy Cheung, Jocelyn Charles, Deepy Sur, Michael Scarpitti, David Willis, Anthony Levitt

**Affiliations:** 1 Sunnybrook Research Institute, Toronto, ON Canada; 2 University of Toronto, Toronto, ON Canada; 3 Sunnybrook Health Sciences Centre, Toronto, ON Canada; 4 Family Advisory Council, Family Navigation Project at Sunnybrook, Toronto, ON Canada; 5 Ontario Association of Social Work, Toronto, ON Canada; 6 Canadian Mental Health Association - Ontario, Toronto, ON Canada; 7 Keystone Child, Youth, and Family Services, Owen Sound, ON Canada

**Keywords:** Transitional-aged youth, Families, Transitions in care, Mental health, Addiction, Access to care, Empowerment, Holistic, Collaborative

## Abstract

**Introduction:**

Transitional-aged youth (TAY) with mental health and/or addictions (MHA) concerns and their families experience significant challenges finding, accessing, and transitioning through needed MHA care. To develop appropriate supports that assist TAY and their families in navigating MHA care, their experiences of transitions in the MHA care system must be better understood. This scoping review identifies and explores the needs, barriers, and facilitators for TAY and their families when transitioning through MHA care.

**Methods:**

This scoping review commenced with a search of five relevant databases. Three research team members were involved in title, abstract, and full-text scanning and data extraction. Sources focusing on TAY anywhere between the ages of 12–29 years and meeting the study objectives were included. Extractions compiled background and narrative information about the nature and extent of the data. Analysis and synthesis of findings involved numerical description of the general information extracted (e.g., numbers of sources by country) and thematic analysis of narrative information extracted (e.g., family involvement in TAY help-seeking).

**Results:**

A total of 5894 sources were identified. Following title and abstract scanning, 1037 sources remained for full-text review. A total of 66 sources were extracted. Findings include background information about extracted sources, in addition to five themes that emerged pertaining to barriers and facilitators to access and transitions through care and the needs and roles of TAY and families in supporting help-seeking and care transitions: holistic supports, proactive preparation, empowering TAY and families, collaborative relationships, and systemic considerations. These five themes demonstrate approaches to care that can ensure TAY and families’ needs are met, barriers are mitigated, and facilitators are enhanced.

**Conclusion:**

This review provides essential contextual information regarding TAY with MHA concerns and their families’ needs when seeking care. Such findings lend to an enhanced understanding of how MHA programs can support this population’s needs, involve family members as appropriate, reduce the barriers experienced, and work to build upon existing facilitators.

**Supplementary Information:**

The online version contains supplementary material available at 10.1186/s12913-023-09430-7.

## Introduction

The transitional-aged youth (TAY) period (typically considered to be anywhere between 12 and 29 years of age) [1, 2] is a time marked by social, developmental, biological, and psychological changes. This also represents a particularly vulnerable time when mental health and addictions (MHA) issues first appear or increase in severity and complexity [2–4]. Early intervention is critical as it may reduce the current and lifetime burden of illness for the youth [1, 2, 5, 6]. For example, in Canada, MHA issues affect an estimated 1.2 million children and youth, yet fewer than 20% receive appropriate treatment for these concerns [6]. TAY have complex needs that impact their transitions through various facets of the care system [7]. As TAY age out of child services, they must meet eligibility criteria for adult services. These transitions can be abrupt, whereby youth may be required to move to the adult system even if they are not ready. This can lead to interruptions in care [4], as TAY may find they are considered not unwell enough or not developmentally ready for adult services, while no longer eligible for child services [4, 8]. TAY also move between multiple levels and types of care, including emergency departments, hospital admissions, outpatient visits, primary care physician visits, and mobile crisis response [9, 10]. They may not receive effective or appropriate treatment for their presenting needs, possibly due to a lack of expertise [11] or lack of access to sufficiently intensive supports [12]. In effect, overall service utilization tends to decrease during care transitions [4] resulting in high levels of unmet need for service and significantly less connection to needed services than other age groups [4, 13]. Experiences of inefficient and unnecessary transitions between services and discontinuous care may lead TAY into developing negative attitudes or mistrust of the system, or into disengaging from care entirely [3, 14, 15].

Family members support TAY by advocating, motivating, and driving the care plan [16], particularly when the youth’s symptoms prevent them from pursuing care on their own [14]. In fact, parental concern for their youth is often the initiating factor for referral to MHA services [14]. Self-referral increases as the youth ages but the family continues to play a significant role, especially until the youth is financially independent [14]. Families’ ability to be a resource for their youth may vary, depending on their own needs and backgrounds [15]. Families are often active in facilitating access to appropriate help, encouraging help-seeking behaviours in their youth, and are the source of important health-related information necessary for providers to complete accurate assessment and monitoring of outcomes [14, 17]. Levels of family involvement vary; some families may wish to encourage independence while remaining available as a “safety net” should the need arise [15]. Involving the family/caregivers can reduce both TAY disengagement from care and caregiver strain [18]. Services can provide parent support in conjunction with youth service, or independently if the youth is not yet willing to engage in service or prefers to receive separate support [2]. Therefore, support for access to and continuity of care for TAY is essential and should include appropriate support for the whole family [11]. Although previous reviews have explored related issues, including: transitions needs of youth with intellectual and developmental disabilities [19, 20], and their families [21, 22] gaps in the Canadian child and adult MHA care systems [23], and even the barriers to care in TAY with MHA concerns; [16, 24] needs, barriers, and facilitators of transitions in care for TAY and their families need to be better understood so that service providers and decision-makers can ensure that TAY and their families experience seamless transitions. Thus, the purpose of this review was to identify and explore the needs of, and barriers and facilitators for, TAY and their families when transitioning through MHA care.

## Methods

Scoping review methodology was used for this study to allow for an examination of the range and extent of literature on the topic of interest, as well as identification of existing gaps in the knowledge base [25]. Scoping reviews also allow for questions that promote greater exploration and conceptual breadth than would be required in systematic reviews [25, 26]. Guidelines from Arksey and O’Malley’s (2005) six-stage framework, further elaborated upon by Levac et al. (2010) were applied in this scoping review. The scoping review protocol was developed a priori and is available on request. The PRISMA Extension for Scoping Reviews Checklist was used to ensure all essential items were captured in this report [27].

### Stage 1: identification of research questions

The research questions guiding this review were developed collaboratively by the research team, which included researchers, medical professionals, MHA system decision-makers, and patient partners. The research questions guiding this review were as follows: (1) What is the role of families in help-seeking activities for TAY with MHA concerns? and (2) What are the barriers and facilitators of access to MHA care for TAY and their families?

### Stage 2: identification of relevant sources

With the assistance of a research librarian at Sunnybrook Health Sciences Centre, a search strategy was developed to identify relevant sources in the following databases: Cochrane Central Register of Controlled Trials (CCRCT), Cochrane Database of Systematic Reviews (CDSR), CINAHL, EmBase, Medline, and PsycINFO. A gray literature search for unpublished research was also conducted using the following sources: Google Advanced, Web of Science, ProQuest Dissertations and Theses, and websites relevant to youth mental health. The inclusion of grey literature was of importance to this study as community-based mental health agencies or other bodies serving TAY might publish reports, guidelines, or patient education materials that address the topic of interest, outside of the academic literature. A combination of relevant key terms was used in the search of both published and gray literature, including and related to TAY, mental health and/or addictions, needs, barriers, facilitators, help-seeking, and families. Results were limited to the English language, and there were no limitations based on the year of publication. Additionally, reference lists of sources chosen for charting were searched by hand to identify relevant sources that were not found through the database searches. See Table 1 for a sample search strategy. Full search strategies for each database are available in Supplementary File 1. Searching was completed on February 11, 2020.

**Table 1:** Sample search strategy (MEDLINE)

#	Search Statement	Results
**1**	exp transition to adult care/	1178
**2**	exp transitional care/	539
**3**	exp Adolescent Health Services/	5386
**4**	*"Continuity of Patient Care”/	9832
**5**	(Transitional Aged Youth* or TAY or emerging adult* or transition to adult* or child to adult or Transition-Age or transitional mental health service* or (young adj3 mental health need*) or youth in transition to adulthood or continuity of care or Transition to Adult Care).mp	15,333
**6**	or/1–5 [TAY]	28,840
**7**	exp Mental Health/	34,417
**8**	exp compulsive behavior/	11,824
**9**	exp Mental Disorders/	1,182,597
**10**	exp Substance-Related Disorders/	267,571
**11**	(mental health or MHA or addict* or Mental Disorder* or substance abuse or Substance-Related Disorder*).mp	470,722
**12**	7 or 8 or 9 or 10 or 11	1,349,745
**13**	exp Developmental Disabilities/	19,145
**14**	exp Intellectual Disability/	93,168
**15**	exp Learning Disorders/	21,544
**16**	exp Autistic Disorder/	19,555
**17**	((Intellectual or developmental or mental or learning or autistic) adj2 (disability* or disorder*)).mp	292,288
**18**	13 or 14 or 15 or 16 or 17	349,614
**19**	12 not 18 [MHA]	1,047,422
**20**	exp Help-Seeking Behavior/	593
**21**	exp Therapeutics/	4,382,979
**22**	exp “Health Services Needs and Demand”/	57,789
**23**	(help-seek* or treatment or Barrier* or difficult* or hinder* or facilitator* or enable or support or care needs or care pathway* or obstacle*).mp	12,790,617
**24**	or/20–23 [HELP]	14,848,386
**25**	6 and 19 and 24	2743
**26**	limit 25 to english language	2656
**27**	remove duplicates from 26	2656

### Stage 3: selection of evidence

**Fig. 1:**
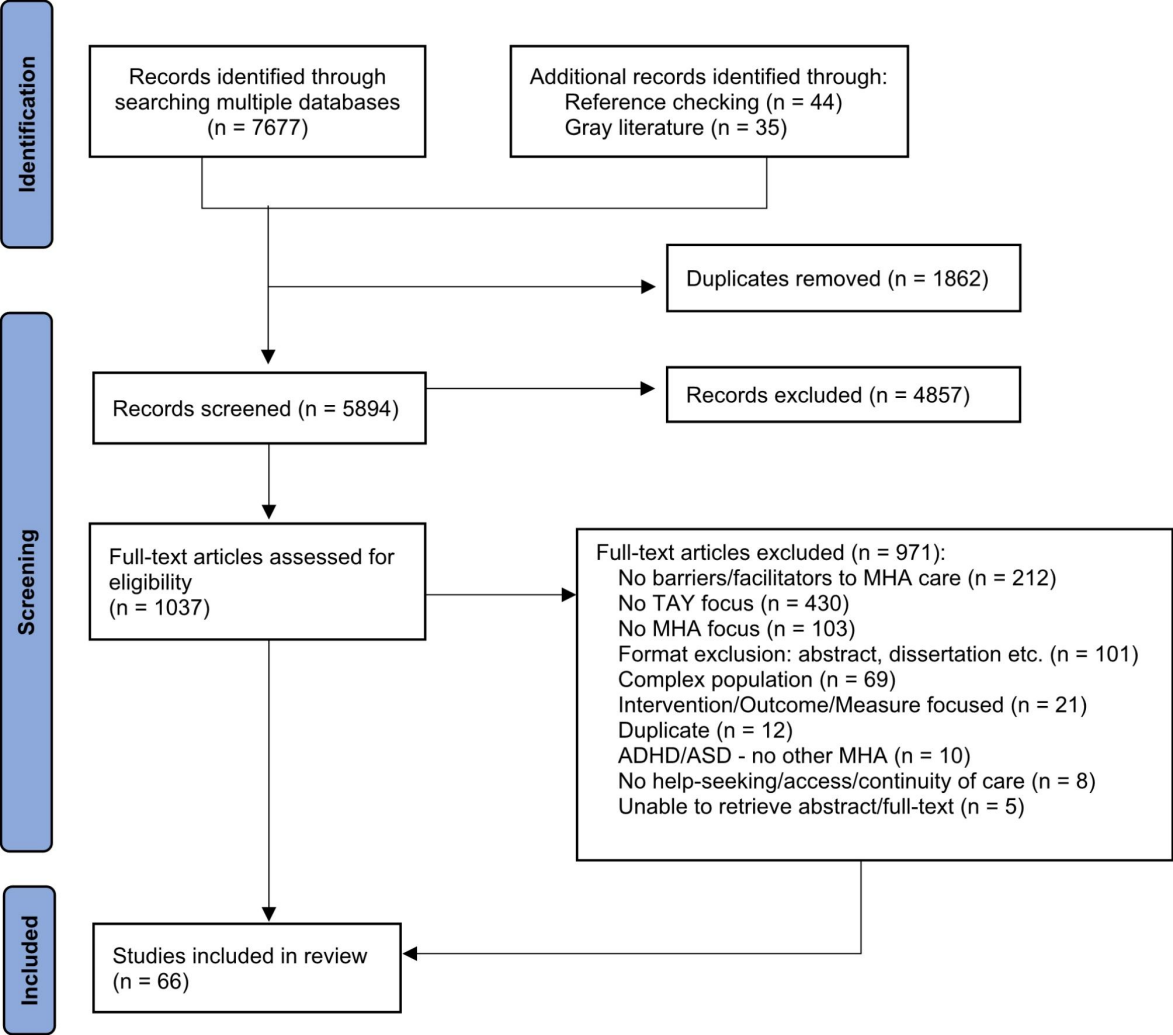
PRISMA flow diagram

Using Covidence software [28], three reviewers individually screened the titles and abstracts of all sources identified through the above search strategy using the following inclusion criteria: sources that focus on factors that help or hinder access to and transitions in mental health and/or addictions care, specifically for transitional aged youth aged 12–29 years; sources that discuss the role of families in MHA care access and transition activities; and sources that examine system perspectives on access to and transitions in care for TAY with MHA concerns. Sources that focused on interventions and/or preventative programs for mental health and/or addictions issues; access primarily for neurodevelopmental disorders; access in relation to complex factors such as homelessness, the justice system, emergency department use, and foster care; and disengagement from treatment were excluded from this review because of the primary focus on access to and transitions in care for mental health and/or addictions issues in TAY. Conference abstracts, dissertations, and book reviews were also excluded from the review. However, other article types (e.g., commentaries, perspectives) were included as they often included narrative reviews, insights from front-line practice, or even first-hand accounts from persons with lived experience; all of which were valuable to integrate into the findings of the current study to supplement learnings from primary research. No exclusions were made based on the year of publication, country of origin, or research methods. As is characteristic of scoping reviews, inclusion/exclusion criteria were refined as familiarity with available literature increased [29]. Full texts of all sources chosen for inclusion were then further screened for eligibility by two members of the research team. A third member of the team was consulted to resolve conflicts related to decisions about source inclusion by meaningfully discussing the rationale for source inclusion. Although generally not required for scoping reviews, a quality assessment rubric was used to confirm a minimum level of quality of each source chosen for inclusion [30]. The rubric utilized was applicable to all article types, and its design and content are reported elsewhere [30]. In general, the rubric supports assessment of various elements of article content (e.g., introduction and aims, sampling, transferability/generalizability), as applicable. This quality appraisal process was undertaken to ensure that sources were appropriately and adequately reported, prior to their inclusion in the review. Two analysts (HC and SC) independently completed the rubric for each source. Following quality appraisal, no sources were removed, in that all obtained a passing score (greater than 18/36). Please see Fig. 1 for the PRISMA flow diagram.

### Stage 4: charting the data

A data extraction form was produced in Microsoft Excel. Two research assistants (HC and SC) charted nine of the same eligible sources to check for consistency and reliability, with review from the study investigator (RM). The categories included in the charting template underwent iterative changes based on findings from charting the initial few sources [29]. The remaining sources were then divided between HC and SC and charted independently, with regular checks for accuracy and consistency by the study investigator (RM). Information charted in the extraction form contributed to the development of a descriptive numerical summary [29] of the sources and included: source objective, study design, sample population characteristics, data collection and analysis methods, findings and discussion related to needs/barriers/facilitators, findings and discussion related to the role of families, conclusions, and strengths/limitations.

### Stage 5: collating, summarizing, and reporting the results

Thematic analysis, a qualitative method to identify, analyze, and organize patterns that emerge from collected data [31], was used to develop themes pertaining to the needs, barriers, facilitators, and role of families identified in the literature. Two members of the research team (HC and SC) independently familiarized themselves with the data by reading and noting down initial thoughts on possible themes, then used MaxQDA software to generate codes based on noteworthy aspects of the data. Frequent collaboration between coders (HC and SC) and with the study lead (RM) during the analysis phase ensured consistency and agreement in code development. These codes were then analyzed and grouped, and similar codes were collapsed to synthesize the findings. RM and HC then independently collated complementary codes to develop several subthemes, which were grouped into a set of broader themes after meeting to refine and clearly define them. For example, codes including ‘need for therapeutic relationships’, ‘lack of human touch’, and ‘abrupt loss of support’ were collated into the subtheme ‘youth-provider relationships’ which was later grouped into the broader theme ‘collaborative relationships’.

### Stage 6: expert consultations

Experts in the field of TAY with mental health and/or addictions issues were identified through the relevant information sources included in this review as well as through discussions of experts known to the research team. A total of six experts were approached and all agreed to participate. While this stage is seen as optional, it is a meaningful opportunity for stakeholder involvement and can also reveal additional literature sources, insights, and knowledge not immediately apparent from the literature surveyed [25]. Considering this as an opportunity for knowledge transfer [29], preliminary findings were shared with stakeholders through a project summary, which included information about the project, data collection, data analysis, and findings, along with a full reference list of all grey and academic sources. These experts were then invited to a one-on-one consultation with either HC or RM, to share their expertise and perspectives, provide feedback on and validate emerging findings, comment on the literature reviewed and whether relevant sources were missing, and provide suggestions on strategies to disseminate findings from the scoping review. Consultations were then coded, thematically analyzed, and integrated with the findings from the literature. After including the information from these consultations, no changes to the themes identified in Stage 5 were needed.

## Results

The electronic database searches produced 7677 articles, out of which 1862 duplicates were removed. Additional records were identified through reference checking (n = 44) and the gray literature search (n = 35). A total of 5894 titles and abstracts were screened. From these, 1037 documents met the inclusion criteria and were retrieved for full-text review. After reviewing full texts, 66 articles were extracted for this scoping review.

### Description of articles

#### Publishing year & geographical location

Most of the articles (n = 55) were published from 2011 onward. Articles mainly originated from the United Kingdom (n = 25), Canada (n = 17), and the United States (n = 10).

#### Study design

54 peer-reviewed articles and 12 Gy literature documents were included in this review. These included, but were not limited to, qualitative studies (n = 18), literature reviews (n = 14), and perspective/commentaries (n = 11) (see Tables 2 and 3). Out of the 66 sources included in this review, 29 explicitly considered the family perspective and/or family needs with regard to MHA transitions in care for TAY, as indicated in the “Family Needs|Roles when Involved” column in Table 3.

**Table 2:** Study design/source type, objectives, key conclusions of reviewed sources

Author Year Country	Study Design/Source Type Objective	Key Conclusions
Abidi 2017 Canada	Perspective/Commentary Consider causes behind the divide between child and adult mental health systems, and outline initiatives attempting to bridge the gaps in service provision for TAY.	Need commitment from all stakeholders; adolescent brain education; transparent early preparation approach; continuous evaluation; elimination of policy-practice gap; and cease determining readiness by age
Appleton 2011 UK	Guide Facilitate development of mental health services for youth.	N/A
Arcelus 2008 UK	Retrospective Compare TAY with eating disorders new to treatment vs. previously treated in CAMHS.	Identified relationship between inpatient admission and low self-esteem, maturational issues
Belling 2014 UK	Qualitative Explore professional perspectives on the barriers and facilitators for CAMHS-AMHS transitions.	Lack of understanding, restrictive criteria, lack of resources all negatively impact CAMHS-AMHS transition and create inequalities
Birleson 2001 Australia	Perspective Commentary supporting the linkage of child and adolescent psychiatry, opposing separate youth mental health programs, and assessing how AMHS can support youth.	- Need to strengthen link between CAMHS and AMHS - Service improvement needed for MH care in TAY - Leaders must take responsibility for closing gaps
Bruce 2008 UK	Literature review Discuss the gap between child and mental health services and suggest ways to bridge it.	Gap can be reduced through collaboration, clearer protocols, and greater understanding between systems
Butterly 2015 Ireland	Perspective Commentary on a personal experience of transition from child to adult mental health services	Lived experience should be clearly communicated and may have impact on MH services
Cappelli 2014 Canada	Quantitative Examine effectiveness of a transition program based on the shared management model.	Shared management model successfully promotes continuity of care during transitions
Children’s Mental Health Ontario 2013 Canada	Literature Review N/A	N/A
Cleverley 2018 Canada	Literature review Identify facilitators of CAMHS-AMHS transitions.	Need to create integrated pathways and care coordination to improve transitions promote youth and family engagement
Davidson 2011 Canada	Policy Examine literature on CAMHS-AMHS transitions, identify evidence for successful transitions, engage stakeholders, and compile knowledge for recommendations on CAMHS-AMHS care approach.	N/A
Davis 2002 USA	Report Describe findings from a survey of experiences of youth and families when transitioning from child to adult mental health services.	Recommendations include improve transition supports, involve all stakeholders, review practices/policies, create anti-stigma campaigns, and research parents’ perspectives.
Davis 2009 USA	Book Chapter N/A	Need for research on currently available services, examination on family involvement during transitions, and evaluation of stakeholder knowledge on TAY strengths/needs
Dimitropoulos 2012 Canada	Qualitative Understand factors/issues/challenges that affect transitions from pediatric to adult eating disorder programs.	- Illness may contribute to disengagement from service and disruption in development; parental involvement reduced during transitions - Future research: larger qualitative study, longitudinal studies, RCTs
Dimitropoulos 2013 Canada	Qualitative Obtain clinician perceptions on barriers/facilitators for effective transitions from pediatric to adult eating disorder program.	Transition should be based on readiness; need to develop model of shared care
Dimitropoulos 2015 (1) Canada	Qualitative Explore experiences of youth with eating disorders who transferred from pediatric to adult care services, specifically focusing on perceptions of parental supports during transitions.	- transitions create conflict between patients and families regarding illness and issues related to emerging adulthood - youth note family can play important role - need to better prepare youth and families for transitions
Dimitropoulos 2015 (2) Canada	Qualitative Examine youth experiences of transitions from pediatric to adult eating disorder programs.	- Need to research how experiences differ among diverse communities - Recommendations to teach life skills and build collaboration between youth and providers
Dowdney 2014 UK	Book chapter Explore continuities and discontinuities in care in various health care systems.	N/A
Dunn 2017 UK	Participatory Describe how a CAMHS Transition Preparation Program was produced.	Methods used were favourable among young people; transition preparation program should include from youth and providers to be engaging and relevant
Embrett 2016 Canada	Literature review Examine literature that assesses existing services for CAMHS-AMHS transitions	Need for better planned transition with collaboration between systems; barriers disrupt flow of transition
Evidence Exchange Network for Mental Health and Addictions 2016 Canada	Literature Review Describe evidence on mental health strategies for TAY.	TAY need integrated system that includes promotion and prevention services
Garland 2019 USA	Case Study Provide context and clinical considerations for facilitating transition into adult mental health care.	Highlights need for transition planning and overlapping care; eating disorders best addressed with an inter-professional team
Gilmer 2012 USA	Qualitative Assess needs/barriers for TAY in youth-specific programs.	Improvement needed in TAY service provision; highlights challenges TAY face when trying to access services
Health Outcomes International 2017 Australia	Environmental analysis N/A	N/A
Hovish 2012 UK	Qualitative Explore experiences of CAMHS users, parents, and professionals on CAMHS-AMHS transitions.	Need to address delayed implementation of youth recommendations despite relevance
Jivanjee 2011 USA	Qualitative Examine focus group data from a larger study on experiences of receiving mental health supports and how they facilitate community integration.	Fragmented system full of challenges; suggests peer support for TAY and parents; need for youth/family collaboration and engagement in treatment and research
Joint Commissioning Panel for Mental Health 2013 UK	Guide Describe what modern transition services should look like.	N/A
Koroloff 1990 USA	Policy Examine how policy can streamline transition services.	Need for system-level change; youth face fragmented, challenging, inaccessible services
Lamb 2013 UK	Perspective Commentary on the complexities involved in improving mental health services for youth.	Need for CAMHS-AMHS collaboration; there is no single solution to improve transitions
Lambert 2014 UK	Perspective Share findings from Peer Support Worker pilot project, which aimed to improve youth experiences through CAMHS-AMHS transitions.	PSW involvement in CAMHS successful
Leavey 2019 UK	Retrospective Evaluate progression through services and associated outcomes, and examine how social aspects of family predict transition outcomes.	Need for engagement and seamless transition
Lindgren 2013 Sweden	Qualitative Examine experiences of professionals on CAP-GenP transitions.	Need for transition planning, provision of information, and cooperation between CAP-GenP
Lindgren 2014 Sweden	Qualitative Explore experiences of young adults and relatives on CAP-GenP transitions.	Need for person-centred planning, consideration of developmental needs; flexibility in transition timing; CAP-GenP joint working
Loos 2018 (1) Germany	Qualitative Examine experiences of TAY service utilization.	Need for staff training on establishing meaningful relationships with TAY; need for interventions to help cope with stigmatizing experiences
Loos 2018 (2) Germany	Qualitative Examine experiences of professionals in CAMHS and AMHS through group discussions.	Need for patient-centred care; need for stable relationships to promote youth engagement; highlights lack of flexibility that contributes to sub-optimal transitions
Mandarino 2014 USA	Literature review Examine barriers faced by TAY when accessing supports external to the child mental health system.	Need for TAY voice in research; policy leaders must be made aware of issues so services can be improved upon
Manuel 2018 USA	Qualitative Explore MH service provider perspectives on the strategies needed to address TAY needs	Need to acknowledge unique TAY needs to improve transition
McDougall 2014 UK	Book Chapter N/A	Need to involve both systems to improve transition outcomes
McGorry 2007 Australia	Perspective N/A	Need for state-level, evidence-informed model development
McGrandles 2012 Scotland	Literature review Identify key issues in CAMHS-AMHS transitions.	Nurses could play important role during transitions; need for provider-patient collaboration for successful transition and consideration of developmental needs
McLaren 2013 UK	Qualitative Explore professional perspectives of the barriers and facilitators to transitions from CAMHS to AMHS.	Cultural divide present between CAMHS and AMHS; need for collaboration, improvement in information transfer
McNamara 2014 Ireland	Qualitative Report findings of a nationwide survey of policies and procedures pertaining to CAMHS-AMHS transitions	Need to prioritize continuity of care for those ineligible for transition, training and collaboration regarding transition
Mental Health Commission of Canada 2017 Canada	Report Inform stakeholders on how to prioritize TAY in mental health funding.	N/A
Mulvale 2015 Canada	Literature review Examine differences in care philosophies between CAMHS and AMHS and how these affect transitions.	AMHS/CAMHS providers need to better understand each other’s care philosophies
Muñoz-Solomando 2010 UK	Literature Review Examine literature on causes for varying quality of adolescent transition services.	Suggested alternate definition of transition; Need for clarity in the field of transition research; Identified principles for effective transition
Paul 2014 UK	Literature review Examine literature on CAMHS-AMHS transitions.	Not enough good quality research on transitional care; Need for research on effectiveness of different models of care for transitions
Plaistow 2014 UK	Literature Review Examine literature on views of young people with regard to barrier and facilitators to engagement in mental health services.	Need to engage young people in service redesign activities
Rayar 2015 Canada	Perspective N/A	Importance of transitions must be addressed
Richards 2004 UK	Qualitative Develop themes of youth mental health services needs based on professional perspectives.	Need for inter-professional forum and protocols, including providers from both child and adult services; need for consistent age thresholds across agencies
Riosa 2015 Canada	Mixed Methods Examine mental health needs of late adolescents.	Youth report moderate self-efficacy, concerns about transition, desire for active participation, and need for trusting relationships
Sainsbury 2011 UK	Report Explore innovative strategies related to CAMHS-AMHS transitions and experiences of youth with mental health problems.	N/A
Salaheddin 2016 UK	Quantitative Examine barriers to accessing mental health support for youth.	Need for improved youth MH literacy, therapeutic relationships to foster help-seeking, stigma reduction, and GP insight into youth MH care
Schandrin 2016 France	Retrospective Analyze quality of transitions from CAMHS to AMHS in a hospital.	CAMHS-AMHS transitions are sub-optimal
Scholz 2019 Australia	Literature review Compile understandings of transition from adolescent to adult mental health services	Need for reformation of transitions for youth MH care
Signorini 2018 Europe - multinational	Quantitative Describe transition policies and highlight gaps in care.	Need to resolve discontinuity of care at transition interface, possibly through common training, shared management, joint review, standardized needs assessment, and active participation in care
Singh 2005 UK	Perspective Commentary on existing barriers to transition and strategies to bridge the divide between child and adult services.	Need for approach that is both ‘top down’ and ‘bottom up’ to improve transition interface
Singh 2010 UK	Mixed methods Present findings from stages 2 and 4 of TRACK study.	Need for evidence-based models of care
Singh 2015 UK	Perspective Commentary on transitions from child to adult mental health services.	Need for urgent service reformation to meet unique needs of young people
Skehan 2017 USA	Literature Review Explore barriers and facilitators in treatment and strategies for TAY.	Need for coordination and continuity of care, family participation, and youth-centred training for providers
Stagi 2015 Italy	Quantitative Evaluate factors related to continuity of care from CAMHS to AMHS.	Need for research on evidence-based treatments and practice for effective transitions
TAYMHA Advisory Committee 2015 Canada	Report N/A	N/A
Ubido 2015 UK	Literature review Present evidence of gaps between child and adult mental health services.	N/A
van der Kamp 2018 UK	Qualitative Describe barriers and facilitators for successful transitions from CAMHS to AMHS.	Need for improvement of transitions
Vloet 2011 Canada	Qualitative Identify evidence for effective transitions and highlight stakeholder perspectives on CAMHS-AMHS transitions.	Need for shared management framework to facilitate effective transitions
Whitney 2012 USA	Perspective Personal experiences of youth with mental health conditions.	Need for services that cater to youth needs and incorporate youth voice
Winston 2012 UK	Perspective Examine differences between CAMHS and AMHS in treating anorexia nervosa.	Need for greater awareness about transition issues and liaisons between services

**Table 3:** Key findings from reviewed sources

Source	Key Findings	
	Needs | Barriers | Facilitators	Family Needs | Roles when Involved
Abidi 2017	■ No coordination between child and adult MH systems ■ Arbitrary age threshold ■ Adolescent feel unprepared for transition ■ Siloing between CAMHS & AMHS ■ Youth prefer structure, active involvement	■ Families feel isolated, helpless in AMHS
Appleton 2011	■ TRACK study highlights poor transitions, awareness of transition barriers ■ No support for continuity of care ■ Arbitrary age threshold ■ Different approaches in CAMHS vs. AMHS ■ No consistent transition protocols	N/A
Arcelus 2008	■ Lack of continuity/coordination of care between child and adult services ■ Problematic age boundaries ■ Insufficient training for AMHS providers	■ Family involvement is part of the gold standard care pathway
Belling 2014	■ Unclear eligibility criteria, inconsistent thresholds ■ High caseloads in AMHS ■ Limited services provided by AMHS	N/A
Birleson 2001	■ Need for link between CAMHS & AMHS to overcome differences	■ CAMHS experience working with families should be shared with AMHS
Bruce 2008	■ Differences, rigid boundaries between CAMHS & AMHS – contributing to traumatizing transitions for youth ■ Limiting eligibility criteria for adult services ■ Insufficient availability of adult services	■ Families feel excluded due to confidentiality concerns in adult services
Butterly 2015	■ Need for youth-centred model, consideration of other transitions	■ Families are given insufficient information regarding youth’s care
Cappelli 2014	■ Transitioned youth have greater ED visits, more unmet needs ■ Varying transition times across AMHS providers	N/A
Children’s Mental Health Ontario 2013	■ Youth fall through the cracks when their illness is deemed not severe enough for service ■ Providers have limited resources to deal with extensive waitlists ■ Barriers present in differences between child and adult care systems, funding structures	N/A
Cleverley 2018	■ Developmental readiness for transition should be considered instead of chronological age ■ Transitions should be formally tracked and managed ■ Transition planning should occur early	■ Family should be involved in entire transition process, from planning to handover of care
Davidson 2011	■ Mismatch between developmental readiness for transition and age cut-offs ■ Need for a youth-centred, inclusive, proactive, collaborative model of care	■ Many youth continue to need family support after transitioning to adult care ■ Families play an important role and should be actively included in youth’s transition care ■ Youth and families should be educated on confidentiality changes during transitions
Davis 2002	■ Parent financial support for services points to severe lack of funding ■ Stigma rated as most common barrier ■ Services deemed often inappropriate for youth age group	■ Many parents expressed frustration at lack of information and feeling excluded from their youth’s care
Davis 2009	■ Variations present in eligibility criteria and age thresholds ■ Differences between child and adult systems further exacerbating transition experiences ■ Fragmentation of funding for MHA care ■ Need for developmentally appropriate services	■ Families often shut out of care after transition into adult system ■ Appropriate level of family involvement can facilitate youth’s treatment progress
Dimitropoulos 2012	■ Denial regarding illness and mixed feelings about recovery posed as significant barriers to active participation in care	■ Parents need to be involved in care regardless of youth’s age ■ Confidentiality is a significant barrier for parent involvement during transition
Dimitropoulos 2013	■ Need for greater flexibility in transition time and consideration for developmental readiness	■ Parent involvement should be gradually decreased and parents should be educated on changing roles
Dimitropoulos 2015 (1)	N/A	■ Need for clarity of family’s role during and after transition
Dimitropoulos 2015 (2)	■ Abrupt loss of support experienced when child MHA care is discontinued ■ Need for conversations about transition well ahead of discharge	■ Conflicts often arise after transitions due to family’s changing role
Dowdney 2014	■ Different eligibility criteria between child and adult systems ■ Poorly coordinated transition protocols	N/A
Dunn 2017	■ Both care systems reported as being not youth-friendly ■ Divide between child and adult care systems ■ Need for joint working between CAMHS and AMHS, especially for transitions	N/A
Embrett 2016	■ Inadequate support available for transitioning youth ■ Siloing of care approaches, funding structures	N/A
Evidence Exchange Network for Mental Health and Addictions 2016	■ Need for reorganization of service delivery, consideration of developmental appropriateness, holistic supports ■ Need for partnerships across services	N/A
Garland 2019	■ Several challenges regarding autonomy pose as barriers to care ■ Many logistical barriers impede transition e.g. transportation, financial costs	■ Facilitated communication between family and youth can strengthen transition and care ■ Need to educate family on how to prepare youth for independence
Gilmer 2012	■ Significant concerns included: long wait times, weak patient-provider relationships, inappropriate level of treatments, inconvenient scheduling ■ Need for more community-based supports and peer mentorship	N/A
Health Outcomes International 2017	■ Youth don’t feel prepared or supported during transitions ■ Divide between CAMHS and AMHS, including funding structures, lack of communication/collaboration ■ Need for self-management skills, active participation of youth in care, joint working between systems	N/A
Hovish 2012	■ Youth felt more prepared and supported when relationship with key worker continued through transition ■ Transition planning meetings considered an important aspect of successful transition ■ Youth experience transitions in other parts of their lives in parallel with transitioning in MHA care	■ Parents experience difficulty adjusting to decrease in involvement after transition ■ Parents preferred greater involvement in care and flexibility in transition time
Jivanjee 2011	■ Youth and parents reported positive experiences when providers were responsive to needs ■ Youth reported ineffective communication with service providers ■ Support groups viewed positively when consisting of participants of the same age	■ Families appreciated wraparound services ■ Families were unhappy with restrictive eligibility criteria, ineffective communication with providers, inaccessible treatment options ■ Youth appreciated parent support post-transition ■ Families appreciated supports from peers in similar situations
Joint Commissioning Panel for Mental Health 2013	■ Divide between CAMHS and AMHS ■ Many youth get lost during transition ■ Absence of good transition protocols	N/A
Koroloff 1990	■ Need for joint planning, early preparation for transition, key transition worker ■ Transition should include planning for other aspects of youth’s life	■ Families should be involved in transition planning
Lamb 2013	■ Training differences between CAMHS and AMHS ■ Youth often have no corresponding AMHS service to transition into ■ Many youth discharged from AMHS without being seen ■ Need for improvement in policy implementation for transitions ■ Need for youth model of transition ■ Need for consideration of developmental age	■ Youth and families want to be actively involved in care ■ Youth and families expressed confusion regarding changes between CAMHS and AMHS
Lambert 2014	■ PSWs facilitate CAMHS-AMHS transitions	■ Families had positive experiences with PSW involvement in transition
Leavey 2019	■ None of the transfers met the criteria for optimal transitions ■ Inconsistent transition protocols	N/A
Lindgren 2013	■ Providers in both systems highlight transition as a time of uncertainty, fear for youth ■ Need for consideration of developmental age ■ Different care approaches in CAMHS vs. AMHS ■ Gaps in service identified by providers in both systems ■ Need for continuation of therapeutic relationship, cooperation between systems, flexibility in transition time	■ Need for good relationship between youth and families
Lindgren 2014	■ Need for consideration of maturity level ■ Transition considered a period of uncertainty, having to start over, loss of secure supports ■ Youth feel left out of care, disruption to care	■ Families understood having to let go, but expressed frustration over not being able to support youth adequately ■ Need for family supports
Loos 2018 (1)	■ Dehumanized care – youth felt unheard and uncared for ■ Need for individualized care, close provider relationships ■ Stigma and passivity as factors influencing health behaviours	■ Desire for ‘parental-like’ support
Loos 2018 (2)	■ Lack of patient-centred care, active participation in care ■ Need for networking between CAMHS & AMHS ■ Need for flexible age boundary	N/A
Mandarino 2014	■ Fragmentation caused by differences between systems results in confusion and disengagement ■ Long wait time for free services ■ Need for both formal and informal support ■ Need for youth involvement in developing programs	■ Support for parental involvement in child system not present in adult system ■ After transition, youth may no longer be considered dependents on parents’ health care plans
Manuel 2018	■ Need for meaningful relationships, support from alumni of treatment, transition worker, proactive planning, active participation in care ■ Conflict between providers and youth on youth wants/needs	■ Family is primary source of motivation, confidence ■ Caregivers experience burnout, exhaustion ■ Level of family engagement depends on quality of family-youth relationship ■ Families also need support through transitions ■ Families responsible for lack of transition readiness
McDougall 2014	■ Need for transition planning recognized previously but still no protocols in place ■ Need for youth voice in transition planning ■ No agreement on age thresholds ■ Transition difficulties impede recovery	■ Desire for family’s active participation in care
McGorry 2007	■ Key barrier: cutting off care at age 18 ■ AMHS insensitive to developmental needs, family needs ■ Need for youth-focused approach ■ Need to increase transition age threshold	N/A
McGrandles 2012	■ Need for agreement on defining transition, consideration of development ■ Differences in CAMHS vs. AMHS in care structures, culture, policies ■ Need for flexible, holistic approach to transition ■ Rigid age thresholds contribute to discontinuous care ■ Cooperation between CAMHS and AMHs facilitates smooth transitions ■ Need for early planning, incorporation of youth voice	■ Strong family ties associated with better MH outcomes – providers can facilitate this by promoting communication between youth and families
McLaren 2013	■ AMHS individual vs. CAMHS family approaches ■ Need for joint working and early communication between systems ■ Need for preparation for transition	■ Transition is difficult for families too ■ Family participation in AMHS care is subject to youth’s wishes
McNamara 2014	■ No proper transition agreements between CAMHS and AMHS, results in unstructured transitions ■ Half of AMHS teams never have a single provider to coordinate transitions ■ Meetings regarding transitions often don’t occur ■ Need for both systems to work together to prepare youth for transition	■ Level of parental involvement varies in AMHS
Mental Health Commission of Canada 2017	■ No coordination between child and adult MH services, differences in cultures, long wait time – barriers to continuity ■ Need for access to equitable care regardless of personal circumstances ■ Need for youth voice in creating solutions ■ Need for flexible, youth-driven, holistic, culturally relevant, empowering, responsive approaches ■ Need for universal training competencies for providers working with youth	■ Need for family-informed approaches, consideration of youth’s circle of care
Mulvale 2015	■ Differences in care approaches, expectations lead to difficult transitions ■ Narrower range of services in AMHS than CAMHS ■ Less follow-up and greater disengagement in AMHS due to emphasis on greater autonomy	■ Decreased family involvement in AMHS, need for youth consent ■ Youth accustomed to family support have greater difficulty transitioning to AMHS
Muñoz-Solomando 2010	■ Need to involve youth and families in planning ■ Differences in care approaches are obstacles during transitions ■ Transitions succeed when providers in child and adults services have good relationships with each other ■ Unique youth needs often not met, greater variation in quality of care within adult system ■ Need for protocols based on best practice, clarity on age thresholds	N/A
Paul 2014	■ Stigma is a barrier to access and engagement ■ Differences between systems disrupt continuity of care ■ Varied transition policies, no specific transition protocols ■ Optimal transition often not experienced	■ Parents desire youth integration into community, preparation for adulthood, solutions for dealing with stigma, peer support, early transition planning, meaningful communication with providers
Plaistow 2014	■ Youth desire information about services: visibility of services, ability to make choices about services ■ Youth desire accessible services: flexible, understandable language, geographically convenient, relaxed atmosphere ■ Desired traits in providers: approachable, genuine, positive, skilled, ability to maintain confidentiality ■ Youth find unhelpful: stigma, lack of information/access, being sent away with medication	N/A
Rayar 2015	■ Mismatch between systems ■ Need for continuity of care ■ Transition viewed as overwhelming, frustrating ■ Having to start over after transition because the systems don’t communicate ■ Not receiving same level of service in the adult system	■ Families less involved in adult systems, increasing risk of youth disengagement from care
Richards 2004	■ Need for consideration of development, personal history, other aspects of youth’s life ■ Need for holistic, flexible approach, greater collaboration between system, key transition worker ■ Insufficient resources lead to long wait lists, inadequate staff ■ Youth considered a minority within both systems ■ Need for youth-friendly, age-appropriate services ■ Difficulties engaging youth due to stigma, reluctance ■ Some youth fall through gaps in care e.g. homeless ■ Need for provider training to meet youth needs ■ Need for consistency in age cut-offs	N/A
Riosa 2015	■ Fear, confusion, ambivalence about transition ■ Desire for right fit with providers, active participation in care	■ Negative experiences with family, communication issues ■ Variability in family involvement, relationships
Sainsbury 2011	■ Youth MH needs are different from children and adults ■ Inconsistent age cut-offs, referral criteria ■ Need for youth’s active participation in care, service planning ■ Need for early transition planning, alternative supports to AMHS, consideration of other life needs, provider collaboration, flexibility, timely provision of information	■ Involve family early
Salaheddin 2016	■ Main stigma barrier: feelings of embarrassment, shame ■ Main attitude barrier: dislike discussing feelings/thoughts ■ Main instrumental barrier: financial costs ■ Misconception about available help as a barrier	■ Fear of speaking up to ask help from family, not wanting to feel like a burden or worry/upset family
Schandrin 2016	■ Youth and families often not included in transition planning ■ Difficulty in collaborating between CAMHS & AMHS due to differences in language, care, structure	N/A
Scholz 2019	■ Successful transitions uncommon ■ Unclear transition pathways ■ Consistent key worker facilitates transition ■ Desire for active participation in care, meaningful patient-provider relationships ■ Insufficient communication between CAMHS & AMHS	■ Decreased parental involvement, decreased access to information ■ Parents reported absence of communication/coordination between providers
Signorini 2018	■ Differences, lack of connection between CAMHS & AMHS ■ Transition planning, teams not common ■ CAMHS case managers not often available; and when they are, they’re often shut out of post-transition care	■ Family involvement dictated by agreement established with youth ■ Family involvement considered part of good transition planning
Singh 2005	■ Child vs. adult psychiatry have different focuses e.g. sociological vs. biological contexts ■ Different perspectives, languages between the systems dictate who can be involved in the care ■ Adolescent developmental aspects overlap with experiences of MHA concerns ■ Rigid age cut-offs ■ More services available in child than adult system ■ Need for training of specialized worker who can facilitate joint working, liaison between systems ■ Need for written transition protocols	■ Families feel excluded from decision-making in adult system
Singh 2010	■ Those with severe illnesses, on medication were more likely to be transitioned ■ Need for transition planning, joint working	■ Parents less involved in AMHS ■ Some youth may not want parents involved in care anymore
Singh 2015	■ Those with less severe concerns are less likely to transition successfully ■ Youth feel unprepared, unsupported ■ Differences between systems weaken transition pathway e.g. culture, organization, funding structure	■ Youth and families feel unheard during transition process ■ Stigma and misperceptions contribute to declining of services
Skehan 2017	■ Developmental phase of rejecting authority may contribute to youth disengaging from care ■ Need for clarity around decision making ■ Need for provider training relevant to youth development ■ Confusion when navigating adult services, need for knowledge/understanding of services ■ Need for age-appropriate services ■ Need for youth voice in program development ■ Need for treatment to focus on transitioning to adulthood rather than adult services	N/A
Stagi 2015	■ Transition more likely for those with more severe concerns ■ Need for collaboration among services	N/A
TAYMHA Advisory Committee 2015	■ Need for consideration of other developmental transitions that complicate youth’s MHA concerns ■ Differences between child and adult systems ■ Need for youth-friendly services, provider training, collaboration across organizations	N/A
Ubido 2015	■ Lack of information available to youth, families, providers ■ Need for transition team to mitigate waitlist issues ■ Need for joint working to overcome differences between systems	■ Families continue to play important roles during transition e.g. advocate, coordinator, nurturer
van der Kamp 2018	■ Inconsistent, short transition periods and outdated protocols ■ Differences between services leave youth unprepared ■ Need for better communication between services ■ Flexible transition age and preparation facilitate transition	■ Youth differ in preference for level of family involvement
Vloet 2011	■ Lack of communication, role confusion at CAMHS-AMHS interface ■ Inflexible funding structures, age thresholds ■ Need for consideration of unique developmental needs, proactive planning	N/A
Whitney 2012	■ Youth experience other life transitions at the same time ■ Age-appropriate supports facilitate transition	N/A
Winston 2012	■ Several differences between CAMHS & AMHS – emphasis on independence, responsibility, level of inpatient treatment ■ Unclear transition procedures, ineffective relationships with providers ■ Need for purposeful transition planning, joint training, proactive approach, consideration of developmental needs	■ Abrupt change in parental involvement – need for gradual transfer of care

#### Sample size

Sample sizes for the qualitative studies included in this review ranged from 8 to 75 participants. Sample sizes in quantitative studies ranged from 203 to 821. For literature reviews included in this review and containing information regarding sample size, samples ranged from 6 to 86 articles.

### Identified themes

Five themes emerged as a result of the thematic analysis of the needs, barriers, and facilitators, as well as the needs and roles of families, in TAY MHA care. These included: holistic supports, proactive preparation, empowering youth and families in transitions, collaborative relationships, and systemic considerations. Each of these themes is named with positive framing, highlighting facilitators that enhance transitions when corresponding supports are appropriately provided. However, there are also barriers for TAY and families related to each theme, when such supports are not provided effectively. As such, identified needs are represented in terms of mitigating barriers and enhancing facilitators. See Table 4 for a matrix depicting themes that arose within the sources reviewed.

**Table 4:** Theme Matrix

Source	Themes				
	Holistic Supports	Proactive Preparation	Empowering Youth & Families	Collaborative Relationships	Systemic Considerations
Arcelus 2008	*	*	*	*	*
Richards 2004	*	*	*	*	*
Muñoz-Solomando 2010	*	*	*	*	*
Birleson 2001	*	*	*	*	*
Loos 2018 (2)	*	*	*	*	*
Singh 2005	*	*	*	*	*
Vloet 2011	*	*	*	*	*
Gilmer 2012	*	*	*	*	*
Lamb 2013	*	*	*	*	*
Butterly 2015	*		*	*	*
Singh 2015	*	*	*	*	*
Embrett 2016	*	*	*	*	*
Singh 2010	*	*	*	*	*
Signorini 2018	*	*	*	*	*
Garland 2019	*	*	*	*	*
Cleverley 2018	*	*	*	*	*
Plaistow 2014	*	*	*	*	*
McNamara 2014		*	*	*	*
Skehan 2017	*	*	*	*	*
Dunn 2017	*	*	*	*	*
Cappelli 2014	*	*	*	*	*
Loos 2018 (1)	*		*	*	*
Scholz 2019	*	*	*	*	*
Whitney 2012	*	*	*		*
Dimitropoulos 2012	*	*	*	*	*
McGrandles 2012	*	*	*	*	*
Dimitropoulos 2013	*	*	*	*	*
Manuel 2018	*	*	*	*	*
Schandrin 2016	*	*	*	*	*
Paul 2014	*	*	*	*	*
Lindgren 2013	*	*	*	*	*
Leavey 2019		*	*	*	*
Koroloff 1990	*	*	*	*	*
McLaren 2013	*	*	*	*	*
Davis 2009	*	*	*	*	*
McDougall 2014	*	*	*	*	*
Mandarino 2014	*	*	*	*	*
Dowdney 2014	*	*	*	*	*
Riosa 2015	*	*	*	*	*
Dimitropoulos 2015 (1)	*	*	*	*	*
van der Kamp 2018	*	*	*	*	*
Jivanjee 2011	*	*	*	*	*
Abidi 2017	*	*	*	*	*
Salaheddin 2016	*	*	*	*	*
Stagi 2015			*	*	*
Belling 2014	*	*	*	*	*
Mulvale 2015	*	*	*	*	*
Hovish 2012	*	*	*	*	*
Bruce 2008	*	*	*	*	*
Winston 2012	*	*	*	*	*
McGorry 2007	*	*	*	*	*
Lindgren 2014	*	*	*	*	*
Lambert 2014	*	*	*	*	*
Dimitropoulos 2015 (2)	*	*	*	*	*
EENet 2016	*	*	*	*	*
Ubido 2015	*	*	*	*	*
Joint Commissioning Panel for Mental Health 2013	*	*	*	*	*
Appleton 2011	*	*	*	*	*
Davis 2002	*	*	*	*	*
Rayar 2015		*	*	*	*
MHCC 2017	*	*	*	*	*
Davidson 2011	*	*	*	*	*
TAYMHA Advisory Committee 2015	*	*	*	*	*
HOI 2017	*	*	*	*	*
CMHO 2013	*	*	*	*	*
Sainsbury 2011	*	*	*	*	*

#### Holistic supports

A comprehensive approach to providing support was emphasized, including flexibility and individualized approaches to transition, considering family needs, and acknowledging the developmental trajectory of TAY while planning for MHA supports. Positive experiences of transitions and access to supports were reported when TAY feel they are being supported and motivated by service providers. In particular, TAY find it helpful when service providers are responsive and available to address their needs and teach them skills to manage their illness [32]. TAY also indicate positive experiences when service providers develop strong rapport with TAY by actively engaging them and giving information and support [33–35]. Feeling supported in these ways is conducive to a positive and interactive environment that allows TAY to open up and share more easily about their MHA concerns [34]. Unfortunately, TAY feel that this kind of support is rarely provided [36], and is too often focused on completing the transfer instead of facilitating transitions for TAY at their own pace [37, 38]. Specifically, several articles noted the need for a holistic approach to address TAY MHA concerns, in that this approach should be flexible in both process and timing; [8, 34, 39–46] individualized to TAY’s needs; [23, 33, 36, 47, 48] and considerate of the family [23, 35, 40–42, 45, 49–52].

The need for flexibility, especially at the point of transfer in TAY’s MHA care journey, was evident. For example, transfer boundaries dictated by age should instead place greater emphasis on the developmental readiness of TAY [38, 43, 44, 49, 51, 53–59], recognizing that services within the adult MHA system are often not developmentally appropriate for TAY [41, 48, 52, 60] and may result in unsuitable treatment plans [58]. Furthermore, lack of flexibility may result in significant gaps in service provision, such as placement on extensive waitlists [56] or even withdrawal from treatment [61]. Presently, many transitions occur as a result of “aging out” of services regardless of TAY’s individual needs [62]. Youth appreciate when service providers defy protocol and allow them to remain within the child MHA system for a few months longer [53]. Parents have been found to share this preference for TAY to remain longer within the child MHA system [63]. These findings speak to the need for service providers to adopt a different approach when working with TAY in the adult MHA system [55, 64]. For example, an ‘age window’ may be appropriate instead of an arbitrary age cut-off [65]. Such approaches would be part of a larger cultural shift needed to acknowledge that TAY are distinct from and differ in needs when compared to younger children and older adults [8]. Flexibility in managing recovery was also suggested, for example, by shifting from a mindset of ‘aging out’ to one of ‘continuing on,’ [64] highlighting that recovery from MHA concerns may be a lifelong journey for many [23, 55, 66] rather than a process with a defined endpoint. Therefore, the timing of transition and the need for flexibility in this regard appear to be important aspects of TAY’s access to MHA care [55].

Because transition often occurs in tandem with a particular age cut-off rather than developmental readiness, continued family involvement throughout the transition process is a strategy for ensuring that TAY do not experience an abrupt loss of support after transitioning in care [35]. Caregivers in particular have requested that information be shared on how they can support TAY during this time [34]. TAY often continue to lean on caregivers for assistance even after they have transitioned in care and are considered adults [52], suggesting the benefit of continued family involvement [40] and caregiver participation in decision-making processes could mitigate TAY feelings of anxiety [67] surrounding transitions. In addition to continued family involvement in TAY’s care journeys, studies indicated a need for holistic supports for families themselves [32, 53, 61, 64], perhaps in the form of peer support, family therapy, and education [49].

Tailored support that focuses on TAY’s strengths and preferences [36, 48, 52, 68] and recognizes their evolving needs and circumstances is also needed [43]. This could include vocational, educational, and housing supports [52], as well as culturally sensitive supports [8, 11, 40, 52, 61, 69] and other specialized services that cater to the goals and needs of TAY [23]. Unfortunately, TAY may encounter a lack of availability of professionals from their own ethnic/cultural group [69]. Cultural sensitivity training for service providers has been proposed to ensure support for diversity in backgrounds, cultures, and languages of TAY [8, 40]. Other life transitions that TAY are experiencing, including changes in school and living situation, should be considered in conjunction with transitions within the MHA system [65, 70, 71] because they may impede TAY access to MHA care [52]. For example, youth, parents, and providers have expressed a need for affordable and age-appropriate housing for youth, highlighting that the current lack of such contributed to increased vulnerability [72]. Thus, TAY require holistic supports that are flexible, considerate of family involvement, and account for developmental needs to facilitate their access to MHA care.

#### Proactive preparation

The literature reviewed identified the need to prepare TAY for transitions in MHA care well ahead of time so that TAY feel ready and know what to expect in these transitions. Appropriate transitions were described as planned, gradual [50, 55], seamless, and proactive [54, 73–75], enabling TAY to be adequately prepared for transition and preventing an abrupt loss of support. Clear and direct conversations with service providers about transition should occur prior to being discharged from a service [8, 34, 57, 76], and may include tours of the next service and meeting with prospective service providers to facilitate a formal handover and continuity of care [37, 38, 50, 56, 57, 63, 73, 76]. These conversations about transitions should occur at the very onset of treatment [64] or at least six months before service termination [34, 38, 41, 45, 56, 77]. Unfortunately, numerous sources identified the absence of transition planning altogether [24, 46, 51, 60, 78, 79].

The pervasive lack of planning was noted as having detrimental effects on TAY, leaving them feeling uncertain [34, 35, 53], frightened [33, 34, 57, 53], vulnerable [23], anxious [34, 80], abandoned [34, 50], and frustrated [35, 81]. One study identified that only a few TAY participants could recall having transfer-related discussions prior to transition [76]. A pervasive absence of formal pathways, guidelines, or protocols to prepare for transition was also noted [8, 24, 37, 46, 55, 79, 80, 82], leading to a disorganized and inconsistent approach to managing transitions [83]. This inconsistency results in transitions that are poorly planned and disjointed [24, 41, 52, 55, 65, 74, 82], with Paul et al [24]. indicating that less than 5% of the young people in their study experienced what they called an ‘optimal transition’. These reactive rather than proactive approaches negatively impact TAY’s care experiences.

Care and funding for TAY has been described as a ‘blind spot’ [44] due to the dearth of clinical resources available to facilitate transitions, mainly due to a lack of funding [8, 24, 44, 47, 49, 58, 60, 61, 67, 69] limiting capacity to provide transition services [24, 39] and effectively prepare TAY for transitions. Service providers often feel limited by insufficient time and resources [34, 55, 65, 67, 71, 80], and high staff turnovers also negatively impact transitions [67]. Overwhelming caseloads resulting from insufficient staffing have been specifically identified as transition barriers [37, 71, 82]. TAY have suggested it is a matter of chance to receive support from a service provider during transitions, such that they may be left without formal support and need to rely on family support during transitions [53]. These features may suggest inadequate prioritization of TAY in the MHA system [8], causing them to fall through care gaps [57, 73]. The absence of transition resources has thus been associated with a tendency for TAY to reject services altogether or feel compelled to settle with care that is inappropriate for their needs [47, 48, 68]. Proactively planning for transitions and allocating the necessary resources for such planning is needed to ease the transition process for TAY in MHA care.

#### Empowering youth and families

The third theme pinpoints the need for TAY and families to feel confident and in control as they experience transitions in their MHA care pathway. TAY and their families need to be empowered in the transition process to feel agency and autonomy in their care, particularly through engagement, education, and mentorship. TAY are often given the responsibility to manage their own care very suddenly when entering the adult system, a task for which they are usually underprepared [52, 74]. Interventions to teach TAY how to confidently and responsibly manage both their illness and their care on their own may facilitate more seamless transitions [33, 34, 43, 49, 56, 69, 76]. Active participation in care may be obstructed by TAY’s denial and/or ambivalence regarding care for MHA concerns [8, 36, 84], possibly arising from a fear of self-advocating [34, 48, 69] in a complex and unfriendly system. Supports should ensure environments conducive to TAY sharing their concerns and needs [58], allowing them to be more active in care and to participate in decisions made about their care before transitions. TAY have expressed a preference for direct conversation with service providers regarding transition prior to being discharged [76], pointing to the need for TAY to be drivers of their treatment [33, 43, 64]. TAY tend to perceive transitions negatively when these conversations do not occur [36, 78], and some TAY may not even be aware that such conversations are possible [52]. These care experiences may leave TAY feeling objectified instead of being considered as individuals with unique care needs [36, 67]. TAY should also be engaged in service redesign, by being encouraged and actively sought to share their views on disengagement from services [39], and what they feel is needed for successful transitions [48]. Engaging TAY in research and program planning in this manner can elicit feedback on how they can best be supported through transitions in care [39, 44, 48, 60, 85]. Empowering TAY by teaching them to manage their care and by facilitating their active participation in care decisions is crucial.

Studies in this review also noted the need for interventions to acknowledge the family’s changing role in TAY’s MHA care. Caregivers need information on changes in policies pertaining to confidentiality as TAY transition into the adult system, as well as information on opportunities to meaningfully care for TAY during these transitions [49, 56, 57, 84]. Caregiver frustration regarding the enforced decrease in their involvement during the transition process has been noted, and caregivers have expressed that TAY independence in care should occur gradually instead of being forced without the proper structural supports in place [53]. The continued involvement of family throughout the transition process is key since they are considered to be important stakeholders and need to be engaged while still supporting increasing TAY independence [32, 44, 45, 47, 50, 86].

Education as a form of empowerment for TAY and families during transitions was also highlighted in the literature reviewed. TAY and families require information on MHA in general, about supports available for MHA concerns, as well as what to expect from such supports. TAY and families need access to information on the supports available to them, with studies noting a misperception of MHA services and unclear expectations regarding the transition process [52, 59, 69, 73, 74, 86], often resulting in a lack of trust and confidence [11, 73] and subsequent reluctance to access care. This can be further mitigated by providing education on the differences between child and adult systems, given service providers in both child and adult systems lack familiarity with each other [45, 48, 62, 67, 70, 71, 87, 88], leading TAY and families lack information on the differences between the two systems [49, 56, 60, 76]. Education was also identified as a strategy to reduce the stigma associated with accessing MHA care [8, 24, 36, 48], which is critical given the considerable care barriers attributable to stigma [11, 33, 35, 39, 60, 64, 66, 69, 73]. The need for mentorship was also highlighted as a mechanism to empower TAY and families in transitions, particularly since alumni of services can provide unique and valuable insights regarding the care that could facilitate access [64, 72]. TAY also find it helpful when they are able to share MHA concerns and experiences with peers who have had similar experiences [8, 52, 89]. Families agree that mentorship is beneficial for TAY and also themselves, including speaking to other parents about navigating the MHA system for TAY [24, 32].

Not only is education important for TAY and families, but it is also necessary to provide training and education to service providers working with TAY in both child and adult systems, specifically with regard to the unique needs of TAY [34, 61, 64]. Limited training opportunities in conjunction with insufficient numbers of service providers who are knowledgeable about transitions [44] contribute to an overall deficiency in providing appropriate care for TAY. In fact, service providers in both the child and adult mental health systems acknowledge they are ill-equipped to work with TAY [8, 44, 49, 74, 77], pointing to the need for child and adult systems to establish familiarity with each other’s care models and take a developmental approach to engaging with TAY, in addition to prioritizing their unique needs and preferences [8, 48, 50, 52, 62, 65, 67, 70, 90]. Overall, empowering TAY and families through engagement, education, and mentorship would support successful transitions.

#### Collaborative relationships

A fourth theme described the relationships among various stakeholders involved in TAY MHA care as a major factor in facilitating access; namely, collaboration between TAY and families with service providers, between service providers, and between TAY and their families. Most importantly, a need for better communication and relationships among all those involved in TAY’s care was highlighted. All stakeholders who have a role in TAY’s transition in care, which includes families, should be identified and roles and responsibilities should be determined early in the care relationship [37, 44, 56, 60, 74, 85]. Input should be provided by all stakeholders, in addition to establishing transparency for all aspects of the care planning process [34, 35, 50, 56]. Furthermore, provider willingness to communicate with families, in addition to open and honest communication among all parties (i.e. TAY, families, and service providers), was identified as foundational for successful transition planning [32, 47]. Studies noted insufficient patient-provider relationships as an area of significant concern [72] with tensions often arising between what service providers believe TAY need and what TAY feel they need [64], resulting in inaccessible care due primarily to the absence of a therapeutic relationship [50, 52, 84]. TAY’s perceptions of their needs and the services they receive may differ significantly from those of service providers [43, 44], and they may perceive service providers as uncaring and pessimistic [32, 34, 36]. In these cases, TAY may feel uncomfortable, unheard, unsupported, and thus unenthusiastic to participate in care [32, 44, 51]. This points to the need for a meaningful and authentic therapeutic relationship between TAY and service providers [36, 38, 48, 53, 67, 72].

Key service provider qualities identified by TAY were approachability, genuineness, and friendliness [32, 36, 38, 39, 82]. TAY reported helpful and supportive experiences when working with service providers possessing such qualities [32, 34, 35, 39, 53], and service providers also agree that establishing positive relationships with TAY is conducive to good care [67]. Several studies also shared the idea of a designated transition worker dedicated to supporting TAY through preparation for transition and bridging the gap between systems [23, 24, 65, 70, 78, 91, 34, 43, 50, 55, 56, 58, 59, 64], for TAY to continue receiving care without interruption or an abrupt loss of support. The importance of a therapeutic relationship with a healthcare professional also arises out of the need for ongoing support and/or monitoring for TAY, to facilitate a smooth transition in care [34, 45, 55] and to reduce the risk of TAY feeling abandoned [50, 57]. Some studies called this a period of parallel care [24, 39, 83] where care is provided before, during, and after transitions by providers in both systems in a joint manner. Without collaborative approaches, termination of support is commonly noted at the transition interface between child and adult systems [23, 59, 53, 73, 74, 76], leaving TAY feeling shocked or overwhelmed about having to start over with a new service [48, 59, 64, 81] and often prompting them to disengage [23, 34, 50, 54, 80] at a time when they need help the most [37]. This results in care fragmentation [24, 47–49, 81, 92], whereby the care pathway is weakened at the point where it needs to be the strongest [73, 75].

Another relationship vital to mitigating the issues of fragmentation discussed above is the one between service providers, specifically collaboration among the various service providers involved with TAY throughout their care journey. Several studies noted that successful transitions in care coincide with good collaboration among service providers [38, 41, 50, 51, 55, 63, 73, 92, 93], especially because this facilitates a formal handover of care that is often lacking for TAY with MHA concerns. However, a lack of communication was apparent among service providers working with TAY, particularly between service providers working within the child system and those working within the adult system [44, 55, 61–63, 71, 74, 78]. This may be due in part to the absence of a common language between systems, lending to poor information transfer and no appropriate forum to discuss issues of crossing over from one service to the next [8, 61, 65, 79, 83]. The absence of communication leads to assumptions, lack of role clarity, and diffusion of responsibility [8, 57, 65, 67, 82]. For example, child system service providers assume that their counterparts within the adult system are not interested in cooperating and collaborating in planning care for TAY [62, 67]. The lack of connection between child and adult systems has been described as the most common difficulty facing TAY [61].

The relationship between TAY and their families is also important for supporting access to MHA care. Many studies highlighted that successful transitions are facilitated by the continuous involvement of family [32, 33, 75, 77, 84, 35, 44, 47, 49, 55, 61, 63, 64] and by service providers’ willingness to communicate with families regarding TAY needs [24, 32, 52, 89]. Families provide valuable support for TAY in the form of motivation, confidence, emotional support, and encouragement in accessing MHA care [32, 64, 86]. The reality, however, is that family involvement is often decreased or eliminated altogether as TAY transition in their care [23, 59, 60, 80, 82], often arising from a push for greater TAY autonomy as they move into the adult system. This can be problematic in circumstances where TAY may be accustomed to relying heavily on family and find themselves expected to abruptly surrender this support, leaving them feeling vulnerable [23, 57] and disengaged from care [81, 84].

In contrast, some studies also reported TAY uncertainty regarding family involvement, with some TAY feeling that families are reluctant to surrender control over their illness and/or recovery [86]. Furthermore, conflict tends to arise regarding the change in the family’s role as TAY transition in care. TAY, families, and service providers may disagree on how involved families should be, often resulting in families feeling shut out of TAY’s care [48, 74, 86]. Issues of confidentiality manifest once TAY reach adulthood, in that TAY may be expected to provide explicit consent for continued family involvement in care. Service providers are restricted from sharing treatment information with families [47, 70], thus limiting the manner in which families can support TAY with MHA concerns [44]. The changes due to confidentiality may be one of the greatest challenges families face during TAY transitions in care [84] because a balance between confidentiality and support can be difficult to achieve [82]. Therefore, collaboration among TAY, families, and service providers is needed to facilitate TAY transitions in care.

#### Systemic considerations

Finally, consideration of systemic components that may contribute to barriers to access to TAY MHA care was highlighted, especially focusing on the appropriateness of services; equity and accessibility; differences between the child and adult MHA systems; the importance of evidence and research on TAY MHA care; and efforts to promote MHA awareness. TAY and families often receive services and/or treatment that is unsuitable to their care needs. Services may not be age-appropriate [8, 60, 66, 79], treatment may not be at the right intensity level, or the treatment modality used may not be the right fit for TAY [39, 55, 72, 76]. Numerous studies recommended approaches for appealing and appropriate services for TAY to effectively engage them in care [45, 47, 52]. A TAY-centred model within existing systems was often identified as ideal in addressing the issue of inappropriate transition support, considering that most current interventions focus either only on children or only on adults while overlooking TAY [42, 52, 66, 92]. For example, social media platforms could be leveraged to provide service information and share options with TAY in a manner more appealing to them [52] and tailored to their needs.

Numerous systemic barriers and restrictions make it difficult for TAY to access care. Financial cost was identified as a common barrier to access, for TAY and families unable to afford services [11, 46, 48, 52, 60, 69]. Additionally, geographical limitations were posed as a barrier to access with studies reporting transportation issues and/or insufficient availability of local services [55, 69]. Admissions criteria for entry into the adult system are often limiting, leaving TAY without support when they have issues considered less severe [71, 82, 87, 93] and/or when services provided in the child system are not mirrored in the adult system [44]. In many cases, there may be no adult service available to match the support received in the child system simply because the child system consists of a different variety of services than the adult system [23, 46, 51, 87]. The mismatch between services in the child and adult systems was identified as a contributing factor to TAY being overlooked and remaining a minority between the two systems [42, 52, 55]. Exacerbating this inattention to TAY needs, participants in several studies also indicated difficulties obtaining information pertinent to accessing care during transitions, causing TAY to feel uninformed and left with little to no help [8, 39, 41, 55, 69, 73]. Furthermore, diagnostic criteria may differ between the systems, meaning TAY who were able to access a support in the child system may no longer be eligible for the same support in the adult system. As a result of these restrictive eligibility criteria to access supports, TAY often find themselves placed on extensive waitlists [34, 39, 55, 54, 72, 76, 77]. Studies also found that the two systems do not share a common language [44, 59, 62, 70] which may result in differing thresholds/criteria for provision of care or lack of recognition of a diagnosis [44], both of which result in inaccessible care for TAY. This points to the need for greater flexibility in eligibility criteria for TAY accessing services, especially during the transition period.

Child and adult systems were also described as differently prioritizing various aspects of TAY’s care. For example, the child system adopts a developmental approach and prioritizes family involvement whereas the adult system approach is more diagnostic and places more emphasis on autonomy [23]. This emphasis on autonomy in the adult system may result in less intense or consistent follow-up for TAY who are now expected to assume sole responsibility for their own care [23, 54], a task for which they are often unprepared. This approach is divergent from what TAY are accustomed to in the child system with its holistic focus and family-oriented approach [42, 50, 52, 55, 84]. These inconsistencies can confuse TAY and families [34, 52], pointing to the great need for transition preparation and collaboration among service providers across systems as discussed above. Moreover, service providers in both systems receive different types of education, training, and funding, consequently establishing different cultures and attitudes, further contrasting treatment approaches between their respective systems [74] and deepening the divide TAY face as they reach the transition boundary [73]. Some studies identified this as the siloing and fragmentation present within the MHA system [74], creating a cultural divide [34, 47, 50, 78] and dissimilar treatment philosophies [23, 24] that together result in inaccessible care for TAY.

Research and evaluation of existing transition programs are needed for quality improvement purposes and to develop appropriate training and/or educational materials for service providers. More precisely, further research needs to be done on how TAY can be best supported through transition because the existing research tends to predominantly focus either on children or adults [52]. Feedback should be sought from TAY, and their caregivers, who are currently transitioning as well as from those who have completed transition to meaningfully assist the development of a TAY-centred transition model [44] and ascertain what best practice for TAY MHA care should look like. This information should subsequently be used to develop formal protocols and/or guidelines to manage transitions [8, 43]. These should also include guidance on recognizing the different approaches between the child and adult systems [23]. Studies noted the gap between policy and practice in this regard, identifying the variability in actual usage of written guidelines [38, 43, 63, 82, 83] and raising concerns about monitoring the implementation of policies developed to address transitions [24, 50].

Lastly, many studies described the presence of stigma toward MHA concerns and highlighted the need for the promotion of MHA awareness as well as stigma reduction. Stigma often prevents TAY and families from engaging with MHA services [8, 24, 35, 39, 48, 60, 64, 69, 73] or contributes to a negative understanding of MHA concerns [36, 39, 53]. Examples of this include feeling ashamed or embarrassed about experiencing MHA concerns [53, 69], fears about being labeled as mentally ill [39, 60] and TAY feeling like a burden on their families [69]. Some studies provided suggestions for reducing the stigma associated with MHA concerns, including establishing MHA awareness campaigns, training service providers regarding MHA promotion, as well as embedding MHA education within school systems [39]. Therefore, on a systemic level, there is a need for appropriate, equitable, and appropriate MHA services; education on the differences between the child and adult MHA systems; research on TAY-centred models of MHA care; and promotion of MHA awareness to facilitate TAY access to MHA care.

## Discussion

This scoping review provides an overview of the needs, barriers, and facilitators encountered by TAY and their families when transitioning through MHA care. The majority of discussion on the topic appeared in the ten years prior to the search being completed, highlighting the recency of awareness of the needs of this particular group of youth and the importance that transitions in care have on the overall care trajectory. Although many sources touched on the role of families in TAY MHA care transitions, the majority overlooked this element of TAY experiences and did not consider family involvement in the care transition experience. Synthesis across all sources identified five key themes that reflect important features of TAY MHA care transitions: holistic supports; proactive preparation; empowering youth and families in transitions; collaborative relationships; and systemic considerations.

TAY need to be considered as unique individuals in their care and supported accordingly, as evidenced across numerous themes found through this review (i.e., holistic supports, proactive preparation, collaborative relationships, empowering youth and families). Youth-centeredness in care is critical, although this is not always evident in child or adult systems that were not designed to focus on TAY. The ambiguous role of family in the youth’s care journey may stem from the lack of focus on TAY, whereby young children are expected to have considerable caregiver/guardian involvement, and adults are expected to be independent. The middle range, in which TAY find themselves, is less clear, and as such, insufficient guidance in this regard exists for providers and for TAY and families. TAY can and should be involved throughout transition planning, a sentiment echoed in studies with and without a focus on MHA care [88, 94]. Many studies have acknowledged the importance of developmental readiness in transitions, for example, by considering emotional and brain development when assessing the capacity to cope with upcoming transitions [95]. The current review revealed that this can and should also include developmental readiness with respect to family involvement, in that TAY may prefer or require their families to remain involved in their care. Family can act as an external motivator for youth, thus aiding them along the care trajectory [96]. However, it is also important to consider TAY preferences regarding family involvement, in that there may be times when family involvement is not the youth’s preference, is not appropriate, or is detrimental.

Regardless of the level of family involvement, evident in this review was the need for support for families in TAY care. Caregivers can experience considerable strain in relation to their TAY’s MHA concerns [97], and may also not be equipped to adapt to their changing role over the course of the TAY’s development and care. Along with TAY, families need to be actively engaged, and supporting families can facilitate TAY engagement in treatment [98, 99]. Caregiver peer support may also be of intrinsic benefit to caregivers themselves [100]. Moreover, this review highlighted the crucial role of providers in collaborating with TAY and families. Providers are uniquely positioned to engage TAY, foster TAY independence, and provide youth-centered care, yet are also tasked with doing so in a manner that acknowledges the TAY’s family dynamic and attitudes toward family involvement. Thus, supporting TAY in an individualized manner, responsive to their preferences and circumstances, is critical in facilitating transitions in care for TAY with MHA concerns and their families.

Considerations related to health equity and the social determinants of health were also evident through this review and the themes that arose (e.g., holistic supports and systemic considerations). To ensure equitable access to care for TAY and families before, during, and following transitions, purposeful efforts are necessary to understand and mitigate the barriers to care that may arise for racialized, marginalized, and vulnerable groups. For example, racialized youth are more likely to experience discrimination in care, face systemic barriers to care (e.g., geographical inaccessibility, lack of insurance), experience cultural differences in experiences of MHA concerns, and are generally less likely than white youth to receive MHA care [101–103]. Similarly, this review highlighted geographic and financial barriers as systemic issues impacting transitions in care that are rooted in the social determinants of health. These factors are also known to impact access to care for youth with MHA concerns in general [101, 104]. Moreover, cultural sensitivity was identified in this review as an important feature of holistic supports, along with the need for focused training in this domain for providers. Existing work has described a lack of culturally competent providers for youth with MHA concerns [101], along with a need for training to challenge implicit biases to reduce disparities in access, ideally through a focus on cultural humility rather than cultural competence [103]. Evidently, systemic efforts are needed to enhance equitable transitions in care and to ensure that once this care is accessed, it is welcoming and appropriate for all TAY in need of MHA care.

A marked need for systemic approaches to transitions in care for TAY with MHA concerns and their families was identified in this review (e.g., proactive preparation, holistic supports, collaborative relationships, and systemic considerations). The reactivity of the MHA care system can be addressed by strengthening system partnerships, enhancing integrated care approaches, and ensuring consistency of support for TAY and their families. Sources in this review indicated clear agreement regarding the current lack of transition planning but with recognition of the importance of transition preparedness. Similarly, a prior review exploring features of successful transitions identified transition readiness and transition planning as two of six core components of transitions, along with policies, monitoring, transfer of care, and transfer completion [56]. In transitions outside of MHA care, the importance of promoting transition readiness by preparing youth ahead of transitions and through structured transition plans has also been noted [105]. The need for flexibility in this process was also evident across sources reviewed, along with comprehensiveness in consideration of family, developmental stage, and other co-occurring life transitions. As such, holistic approaches to transitions, such that they occur gradually while keeping individualized needs at the fore, would greatly benefit TAY and families [98, 106].

Correspondingly, in systems outside of MHA care, later transitions have been shown to improve outcomes and satisfaction [107]. Unfortunately, as identified in this review, the pervasiveness of resource needs is a significant barrier to proactively preparing youth for transition. Providers are overburdened and unable to take on these additional roles. These limitations point to the importance of clear identification of system issues that are preventing effective transitions in care and purposeful, responsive funding of these priorities. TAY and families may rely on their primary care providers when experiencing MHA concerns and during transitions, as they have pre-existing relationships when these concerns arise. However, primary care providers experience difficulties identifying and connecting TAY and families with appropriate resources and are desirous of better connections with MHA supports [108]. Another possibility is navigation support, which can help act as a bridge across systems and services [98]. Navigators spend significant time learning about available resources in the system, along with learning about TAY and families’ individual needs, to make the most appropriate matches to care and support TAY and families along the care trajectory [109, 110]. Navigation may enhance all themes identified in this review by providing holistic supports; enabling proactive preparation; empowering youth and families in transitions; supporting collaborative relationships; and responding to and informing systemic considerations [111]. In this manner, navigation, and other related integrated care solutions, have considerable potential to optimize transitions in care for TAY with MHA concerns and their families.

There are a number of important limitations to consider in this scoping review. Firstly, MHA concerns and needs often co-occur with other health, developmental, or social needs and consequently, transitions arising from these needs. To manage the scope of this review, articles pertaining to other health conditions, developmental disabilities, or social systems (e.g., transitions out of foster care) were excluded. However, literature regarding transitions in care in these populations may hold important information regarding the intersections of these needs with transitions in MHA care. These intersections can and should be purposefully explored in future work. Furthermore, this scoping review focuses on literature published before the COVID-19 pandemic. As such, it does not account for the sweeping systemic changes and pressures from overwhelming mental health needs among youth and families during the past two years. It is anticipated that the identified needs have only been exacerbated rather than resolved, and focused primary research or literature reviews exploring the experiences of TAY and their families during the pandemic timeframe are warranted.

## Conclusion

Despite the substantial evidence base identified pertaining to transitions in care for TAY with MHA concerns and their families, the need for improvement in care experiences was apparent. The importance of supported transitions was clearly evident, and centered on the implementation of holistic supports, proactive preparation, empowering youth and families, collaborative relationships, and systemic considerations. Also demonstrated in this review was that the creation of space for appropriate family involvement must be intentional, recognizing family as crucial care partners while also enabling the TAY’s development and autonomy. Future work may purposefully explore the role of the family, including when family involvement is appropriately indicated, and develop guidance for providers accordingly. Integrating the themes found through this study will help ensure youth- and family-centered policies and practices that prevent TAY experiences of inefficient and ineffective transitions in care. This review has identified and explored critical considerations in the support of TAY with MHA concerns and their families, thereby illustrating key approaches to ensuring these youth and families experience optimal transitions in care.

## Electronic supplementary material

Below is the link to the electronic supplementary material.

Supplementary Material 1: Search Strategies

## Data Availability

Available upon reasonable request from the corresponding author.

## List of abbreviations

CCRCT: Cochrane Central Register of Controlled Trials
CDSR: Cochrane Database of Systematic Reviews
CINAHL: Cumulative Index to Nursing and Allied Health Literature
MHA: Mental health and/or addictions
PRISMA: Preferred reporting items for systematic reviews and meta-analyses
TAY: Transitional-aged youth
