# Supplementary material for: Transitions in mental health and addiction care for youth and their families: a scoping review of needs, barriers, and facilitators
Source: BMC Health Serv Res. 2023 May 10;23:470. doi: 10.1186/s12913-023-09430-7 (PMC10171912; doi:10.1186/s12913-023-09430-7)
Supplement: Supplementary file 1 — Supplementary Material 1: Search Strategies [file 12913_2023_9430_MOESM1_ESM.docx]

Supplementary File 1: Search Strategies

Database: EBM Reviews - Cochrane Central Register of Controlled Trials <June 2019>

Search Strategy:

--------------------------------------------------------------------------------

1 exp Transition to Adult Care/ (15)

2 exp Adolescent Health Services/ (166)

3 exp "Continuity of Patient Care"/ (608)

4 (Transitional Aged Youth* or TAY or emerging adult* or transition to adult* or child to adult or Transition-Age or

transitional mental health service* or (young adj3 mental health need*) or youth in transition to adulthood or

continuity of care or Transition to Adult Care).mp. [mp=title, original title, abstract, mesh headings, heading words,

keyword] (945)

5 or/1-4 [TAY] (1600)

6 exp Mental Health/ (1278)

7 exp Compulsive Behavior/ (611)

8 exp Mental Disorders/ (61888)

9 exp Substance-Related Disorders/ (13629)

10 exp Behavior, Addictive/ (536)

11 (mental health or MHA or addict* or Mental Disorder* or substance abuse or Substance-Related Disorder*).mp.

[mp=title, original title, abstract, mesh headings, heading words, keyword] (35156)

12 exp Developmental Disabilities/ (567)

13 exp Intellectual Disability/ (1264)

14 exp Learning Disorders/ (566)

15 exp Autistic Disorder/ (848)

16 ((Intellectual or developmental or mental or learning or autistic) adj2 (disability* or disorder*)).mp.

[mp=title, original title, abstract, mesh headings, heading words, keyword] (14212)

17 6 or 7 or 8 or 9 or 10 or 11 (84267)

18 12 or 13 or 14 or 15 or 16 (15396)

19 17 not 18 [MHA] (71941)

20 exp "Health Services Needs and Demand"/ (428)

21 exp Therapeutics/ (280985)

22 exp Critical Pathways/ (181)

23 (help-seek* or treatment or Barrier* or difficult* or hinder* or facilitator* or enable or support or care needs

or care pathway* or obstacle*).mp. [mp=title, original title, abstract, mesh headings, heading words, keyword] (760783)

24 or/20-23 [HELP] (863138)

25 5 and 19 and 24 (209)

26 1 or 4 (945)

27 19 and 24 and 26 (116)

28 limit 27 to english language (81)

29 remove duplicates from 28 (76)

Database: EBM Reviews - Cochrane Database of Systematic Reviews <2005 to July 10, 2019>

Search Strategy:

--------------------------------------------------------------------------------

1 (Transitional Aged Youth* or TAY or emerging adult* or (transition adj2 adult*) or child to adult or

Transition-Age or transitional mental health service* or (young adj2 mental health need*) or youth transition to

adulthood or Transition to Adult Care).mp. [mp=title, abstract, full text, keywords, caption text] (137)

2 (mental health or MHA or addict* or Mental Disorder* or substance abuse or Substance-Related Disorder*).mp.

[mp=title, abstract, full text, keywords, caption text] (1736)

3 ((Intellectual or developmental or mental or learning or autistic) adj2 (disability* or disorder*)).mp. [mp=title,

abstract, full text, keywords, caption text] (850)

4 2 not 3 (986)

5 (help-seek* or treatment or Barrier* or difficult* or hinder* or facilitator* or enable or support or care needs

or care pathway* or obstacle*).mp. [mp=title, abstract, full text, keywords, caption text] (10579)

6 1 and 4 and 5 (20)

7 remove duplicates from 6 (20)

Database: EBSCOhost Research Databases Search Screen – Advanced Search Database – CINAHL <July 2019>

Search Strategy:

| **Search ID#** | **Search Terms** | **Search Options** | **Results** |
| --- | --- | --- | --- |
| S31 | s27 not s30 | Search modes - Boolean/Phrase | 1,564 |
| S30 | S28 OR S29 | Search modes - Boolean/Phrase | 129,023 |
| S29 | hiv or aids or acquired human immunodeficiency syndrome or human immunodeficiency virus | Search modes - Boolean/Phrase | 128,880 |
| S28 | (MH "Human Immunodeficiency Virus+") | Limiters - English Language  Search modes - Boolean/Phrase | 8,449 |
| S27 | S5 AND S19 AND S25 | Limiters - English Language  Search modes - Boolean/Phrase | 1,645 |
| S26 | S5 AND S19 AND S25 | Search modes - Boolean/Phrase | 1,663 |
| S25 | S20 OR S21 OR S22 OR S23 OR S24 | Search modes - Boolean/Phrase | 1,393,490 |
| S24 | help-seek* or treatment or Barrier* or difficult* or hinder* or facilitator* or enable or support or care needs or care pathway* or obstacle* | Search modes - Boolean/Phrase | 1,376,404 |
| S23 | (MH "Critical Path") | Search modes - Boolean/Phrase | 4,830 |
| S22 | (MH "Health Services Needs and Demand+") | Search modes - Boolean/Phrase | 22,541 |
| S21 | (MH "Attitude to Medical Treatment") | Search modes - Boolean/Phrase | 511 |
| S20 | (MH "Help Seeking Behavior") | Search modes - Boolean/Phrase | 5,720 |
| S19 | S11 not S18 | Search modes - Boolean/Phrase | 461,349 |
| S18 | S13 OR S14 OR S15 OR S16 OR S17 | Search modes - Boolean/Phrase | 120,277 |
| S17 | (Intellectual or developmental or mental or learning or autistic) N2 (disability* or disorder*) | Search modes - Boolean/Phrase | 109,592 |
| S16 | (MH "Autistic Disorder") | Search modes - Boolean/Phrase | 20,343 |
| S15 | (MH "Learning Disorders+") | Search modes - Boolean/Phrase | 8,837 |
| S14 | (MH "Mentally Disabled Persons") | Search modes - Boolean/Phrase | 4,284 |
| S13 | (MH "Developmental Disabilities") | Search modes - Boolean/Phrase | 8,546 |
| S12 | (MH "Intellectual Disability+") | Search modes - Boolean/Phrase | 27,474 |
| S11 | S6 OR S7 OR S8 OR S9 OR S10 | Search modes - Boolean/Phrase | 575,512 |
| S10 | mental health or MHA or addict* or Mental Disorder* or substance abuse or Substance-Related Disorder* | Search modes - Boolean/Phrase | 211,006 |
| S9 | (MH "Substance Abuse+") OR (MH "Substance Dependence+") | Search modes - Boolean/Phrase | 117,676 |
| S8 | (MH "Mental Disorders+") | Search modes - Boolean/Phrase | 493,580 |
| S7 | (MH "Behavior, Addictive+") | Search modes - Boolean/Phrase | 86,034 |
| S6 | (MH "Mental Health") | Search modes - Boolean/Phrase | 30,056 |
| S5 | S1 OR S2 OR S3 OR S4 | Search modes - Boolean/Phrase | 32,809 |
| S4 | Transitional Aged Youth* or TAY or emerging adult* or transition to adult* or child to adult or Transition-Age or transitional mental health service* or (young N3 (mental health need*)) or youth in transition to adulthood or continuity of care or Transition to Adult Care | Search modes - Boolean/Phrase | 13,528 |
| S3 | (MH "Adolescent Health Services") | Search modes - Boolean/Phrase | 2,565 |
| S2 | (MH "Continuity of Patient Care+") | Search modes - Boolean/Phrase | 16,800 |
| S1 | (MH "Transitional Programs") | Search modes - Boolean/Phrase | 2,614 |

Database: Embase Classic+Embase <1947 to 2019 Week 28>

Search Strategy:

--------------------------------------------------------------------------------

1 exp transition to adult care/ (1642)

2 exp transitional care/ (2219)

3 (Transitional Aged Youth* or TAY or emerging adult* or transition to adult* or child to adult or Transition-Age or

transitional mental health service* or (young adj3 mental health need*) or youth in transition to adulthood or

continuity of care or Transition to Adult Care).mp. [mp=title, abstract, heading word, drug trade name, original title,

device manufacturer, drug manufacturer, device trade name, keyword, floating subheading word, candidate term word]

(21173)

4 or/1-3 [TAY] (23147)

5 exp mental health/ (147520)

6 exp addiction/ (304990)

7 exp substance abuse/ (52084)

8 (mental health or MHA or addict* or Mental Disorder* or substance abuse or Substance-Related Disorder*).mp.

[mp=title, abstract, heading word, drug trade name, original title, device manufacturer, drug manufacturer, device trade

name, keyword, floating subheading word, candidate term word] (504722)

9 exp developmental disorder/ (41708)

10 exp intellectual impairment/ (508783)

11 exp learning disorder/ (36050)

12 exp autism/ (64031)

13 ((Intellectual or developmental or mental or learning or autistic) adj2 (disability* or disorder*)).mp. (173011)

14 9 or 10 or 11 or 12 or 13 (694961)

15 5 or 6 or 7 or 8 (683327)

16 15 not 14 [MHA] (579739)

17 exp help seeking behavior/ (10332)

18 therapy/ (1359493)

19 exp mental health service/ (57065)

20 exp health care need/ (27930)

21 (help-seek* or treatment or Barrier* or difficult* or hinder* or facilitator* or enable or support or care needs

or care pathway* or obstacle*).mp. (8835979)

22 or/17-21 [HELP] (9666438)

23 4 and 16 and 22 (1581)

24 limit 23 to english language (1523)

25 remove duplicates from 24 (1502)

Database: Ovid MEDLINE(R) and Epub Ahead of Print, In-Process & Other Non-Indexed Citations, Daily and Versions(R) <1946

to July 18, 2019>

Search Strategy:

--------------------------------------------------------------------------------

1 exp transition to adult care/ (1178)

2 exp transitional care/ (539)

3 exp Adolescent Health Services/ (5386)

4 *"Continuity of Patient Care"/ (9832)

5 (Transitional Aged Youth* or TAY or emerging adult* or transition to adult* or child to adult or Transition-Age or

transitional mental health service* or (young adj3 mental health need*) or youth in transition to adulthood or

continuity of care or Transition to Adult Care).mp. [mp=title, abstract, original title, name of substance word, subject

heading word, floating sub-heading word, keyword heading word, organism supplementary concept word, protocol

supplementary concept word, rare disease supplementary concept word, unique identifier, synonyms] (15333)

6 or/1-5 [TAY] (28840)

7 exp Mental Health/ (34417)

8 exp compulsive behavior/ (11824)

9 exp Mental Disorders/ (1182597)

10 exp Substance-Related Disorders/ (267571)

11 (mental health or MHA or addict* or Mental Disorder* or substance abuse or Substance-Related Disorder*).mp.

[mp=title, abstract, original title, name of substance word, subject heading word, floating sub-heading word, keyword

heading word, organism supplementary concept word, protocol supplementary concept word, rare disease supplementary

concept word, unique identifier, synonyms] (470722)

12 7 or 8 or 9 or 10 or 11 (1349745)

13 exp Developmental Disabilities/ (19145)

14 exp Intellectual Disability/ (93168)

15 exp Learning Disorders/ (21544)

16 exp Autistic Disorder/ (19555)

17 ((Intellectual or developmental or mental or learning or autistic) adj2 (disability* or disorder*)).mp.

[mp=title, abstract, original title, name of substance word, subject heading word, floating sub-heading word, keyword

heading word, organism supplementary concept word, protocol supplementary concept word, rare disease supplementary

concept word, unique identifier, synonyms] (292288)

18 13 or 14 or 15 or 16 or 17 (349614)

19 12 not 18 [MHA] (1047422)

20 exp Help-Seeking Behavior/ (593)

21 exp Therapeutics/ (4382979)

22 exp "Health Services Needs and Demand"/ (57789)

23 (help-seek* or treatment or Barrier* or difficult* or hinder* or facilitator* or enable or support or care needs

or care pathway* or obstacle*).mp. [mp=title, abstract, original title, name of substance word, subject heading word,

floating sub-heading word, keyword heading word, organism supplementary concept word, protocol supplementary concept

word, rare disease supplementary concept word, unique identifier, synonyms] (12790617)

24 or/20-23 [HELP] (14848386)

25 6 and 19 and 24 (2743)

26 limit 25 to english language (2656)

27 remove duplicates from 26 (2656)

Database: PsycINFO <1987 to July Week 3 2019>

Search Strategy:

--------------------------------------------------------------------------------

1 exp client transfer/ (239)

2 exp Adolescent Health/ and exp Mental Health Services/ (15)

3 exp "Continuum of Care"/ (1669)

4 (Transitional Aged Youth* or TAY or emerging adult* or transition to adult* or child to adult or Transition-Age or

transitional mental health service* or (young adj3 mental health need*) or youth in transition to adulthood or

continuity of care or Transition to Adult Care).mp. [mp=title, abstract, heading word, table of contents, key concepts,

original title, tests & measures, mesh] (11428)

5 or/1-4 [TAY] (12613)

6 exp Mental Health/ (55896)

7 exp Mental Disorders/ (719220)

8 exp Drug Abuse/ (44046)

9 exp Addiction/ (24875)

10 (mental health or MHA or addict* or Mental Disorder* or substance abuse or Substance-Related Disorder*).mp.

[mp=title, abstract, heading word, table of contents, key concepts, original title, tests & measures, mesh] (351029)

11 exp Developmental Disabilities/ (13212)

12 exp Intellectual Development Disorder/ (27146)

13 exp Learning Disorders/ (24387)

14 exp Autism Spectrum Disorders/ or exp Cognitive Impairment/ (72289)

15 ((Intellectual or developmental or mental or learning or autistic) adj2 (disability* or disorder*)).mp.

[mp=title, abstract, heading word, table of contents, key concepts, original title, tests & measures, mesh] (145937)

16 6 or 7 or 8 or 9 or 10 (882554)

17 11 or 12 or 13 or 14 or 15 (234488)

18 16 not 17 [MHA] (667393)

19 exp help seeking behavior/ (11251)

20 exp Treatment/ or exp Treatment Barriers/ (861618)

21 exp health service needs/ (5581)

22 (help-seek* or treatment or Barrier* or difficult* or hinder* or facilitator* or enable or support or care needs

or care pathway* or obstacle*).mp. [mp=title, abstract, heading word, table of contents, key concepts, original title,

tests & measures, mesh] (1136283)

23 or/19-22 [HELP] (1516839)

24 5 and 18 and 23 (1929)

25 limit 24 to english language (1839)

26 remove duplicates from 25 (1837)
